# Interval training suppresses nod-like receptor protein 3 inflammasome activation to improve cardiac function in myocardial infarction rats by hindering the activation of the transforming growth factor-β1 pathway

**DOI:** 10.1186/s13019-024-02756-1

**Published:** 2024-05-10

**Authors:** Wei Wei, Ping Xie, Xuemei Wang

**Affiliations:** 1 Cardiovascular medicine, Zhangye Second People’s Hospital, North Section of West 3rd Ring Road, Binhe New District, Ganzhou District, Zhangye, 734000 China; 2 Cardiovascular medicine, Gansu Provincial Hospital, Lanzhou, China

**Keywords:** Myocardial infarction, Interval training, TGF-β1, NLRP3, Oxidative stress, Cardiac function

## Abstract

**Objective:**

Myocardial infarction (MI) -induced cardiac dysfunction can be attenuated by aerobic exercises. This study explored the mechanism of interval training (IT) regulating cardiac function in MI rats, providing some theoretical basis for clarifying MI pathogenesis and new ideas for clinically treating MI.

**Methods:**

Rats were subjected to MI modeling, IT intervention, and treatments of the Transforming growth factor-β1 (TGF-β1) pathway or the nod-like receptor protein 3 (NLRP3) activators. Cardiac function and hemodynamic indicator alterations were observed. Myocardial pathological damage and fibrosis, reactive oxygen species (ROS) level, superoxide dismutase (SOD), catalase (CAT) and glutathione peroxidase (GSH-Px) activities, MDA content, inflammasome-associated protein levels, and inflammatory factor levels were assessed. The binding between TGF-β1 and receptor was detected.

**Results:**

MI rats exhibited decreased left ventricle ejection fraction (LVEF), left ventricle fractional shortening  (LVFS), left ventricular systolic pressure  (LVSP), positive and negative derivates max/min (dP/dt max/min) and increased left ventricular end-systolic pressure (LVEDP), a large number of scar areas in myocardium, disordered cell arrangement and extensive fibrotic lesions, increased TGF-β1 and receptor binding, elevated ROS level and MDA content and weakened SOD, CAT and GSH-Px activities, and up-regulated NLRP3, apoptosis-associated speck-like protein containing a CARD  (ASC) and cleaved-caspase-1 levels, while IT intervention caused ameliorated cardiac function. IT inactivated the TGF-β1 pathway to decrease oxidative stress in myocardial tissues of MI rats and inhibit NLRP3 inflammasome activation. Activating NLRP3 partially reversed IT-mediated improvement on cardiac function in MI rats.

**Conclusion:**

IT diminished oxidative stress in myocardial tissues and suppressed NLRP3 inflammasome activation via inactivating the TGF-β1 pathway, thus improving the cardiac function of MI rats.

**Supplementary Information:**

The online version contains supplementary material available at 10.1186/s13019-024-02756-1.

## Introduction

Myocardial infarction (MI) stands out as one of the cardiovascular diseases (CVD) with the highest incidence rate and mortality globally, with its morbidity rising rapidly along with aging [1]. As the population of global elderly grows, the significance of MI prevention and treatment has further increased, and as a result, effective intervention measures are urgently needed for adjuvant therapy [2].

As is known to all, appropriate exercise training plays a crucial role in cardiovascular function improvement for cardiovascular patients and healthy people [3–5]. Actually, aerobic exercise has the ability to upgrade endothelial and vasomotor functions in CVD patients, thereby improving myocardial blood flow [6]. What’s more, accumulating evidence has demonstrated that exercise training may contribute to changing MI-caused myocardial remodeling and ameliorating cardiac function [7–10]. Notably, interval training (IT) has been confirmed to have more benefits in elevating peak oxygen uptake and oxygen among different aerobic exercise modes in patients with CVD [11]. Currently, research has shown that IT is capable of augmenting cardiovascular health, but the specific regulatory mechanism remains unclear. Myocardial ischemia stands out as the main cause of MI, encouraging the overexpression of reactive oxygen species (ROS) in ischemic and surrounding myocardium [12, 13]. The overexpression of ROS post-MI exacerbates the oxidative stress reaction and the production of inflammatory cytokines, which then damages the myocardial membrane and causes ischemic necrosis and apoptosis of cardiomyocytes [14]. Myocardial ischemic injury will further trigger pathological cardiac remodeling, including compensatory hypertrophy of myocardial cells and myocardial fibrosis in the surrounding area of the infarction [15, 16]. Remodeling of these myocardial cells deteriorates heart function, ultimately leading to cardiac dysfunction and heart failure [17]. Due to the essential role of oxidative stress in left ventricular remodeling after MI, its inhibition has become the main therapeutic target for the mitigation of pathological remodeling and systolic dysfunction after MI [18]. Transforming growth factor-β1 (TGF-β1), a crucial regulatory factor for cardiac fibrosis, possesses varying function, such as affecting cell growth, apoptosis, and differentiation, increasing the production of collagen and matrix protein, maintaining fibroblast viability, as well as suppressing the production of metalloproteinases that facilitates collagen degradation [19, 20]. Significantly, high expression of TGF-β1 is observed in MI and blockage of the TGF-β1 pathway can alleviate myocardial fibrosis in MI mouse models [21]. TGF-β family receptor is part of the serine/threonine kinase family, in which the type II receptor activates the kinase activity of type I receptor through phosphorylating its near membrane region [22]. Guanxin V efficiently mitigates cell apoptosis, fibrosis, oxidative stress damage via down-regulating the TGF-β1 pathway, thereby mitigating MI [23]. Moreover, exercise training is able to modulate the TGF-β1-Smad2/3-MMP2/9 pathway to diminish oxidative stress, myocardial fibrosis, and cell apoptosis, thus ameliorating myocardial function [24]. The nod-like receptor protein 3 (NLRP3) inflammasomes, as a participant of inflammatory immune response, correlates closely with CVD [25]. A previous study has attestedthat the NLRP3 inflammasomes are involved in the emergence and development of diseases including cardiomyopathy, ischemia-reperfusion injury (MIRI), arrhythmia [26]. In MIRI, pubescenoside A restrains the activation of oxidative stress-initiated NLRP3 inflammasomes via the covalent modification of Keap1 at cysteine (Cys)77 and Cys434 [27]. Consisting of caspase-1, NLRP3 and apoptosis-associated speck-like protein containing a CARD (ASC), the multi-protein inflammatory complex has a close relationship with MI [28]. Additionally, alleviation of NLRP3-mediated inflammation and diminution of pro-inflammatory cytokine expression leads to reduced MI area and mitigated myocardial tissue remodeling, ultimately protecting cardiac function [29]. Nevertheless, it remains unknown whether IT can inhibit the activation of NLRP3 inflammasomes by inactivating the TGF-β1 pathway, thereby improving cardiac function in MI rats. Therefore, the purpose of this study was to explore the mechanism, which provided partial theoretical basis for clarifying the pathogenesis of MI and new ideas for treating MI.

## Materials and methods

### Ethics statement

Animal experiments were authorized by the academic ethics committee of Zhangye Second People’s Hospital. All procedures were strictly implemented by the laboratory animal management and use regulations. We had done our utmost to reduce the number of animals used and alleviate their suffering.

### Experimental animals

We acquired 80 normal male Sprague-Dawley rats weighing 220–250 g, and the rats were provided by Experimental Animal Center of Lanzhou University (Lanzhou, China). They were raised with standard diet and water in a 12-h dark/light cycle, at 20 ± 2 °C with a humidity of 50 ± 2%.

### Animal modeling

The MI rat model was established using the left anterior descending artery (LAD) ligation method [30, 31]. Briefly, all rats were anesthetized via an intraperitoneal injection with 5% pentobarbital sodium at a dosage of 30 mg/kg, followed by ventilation using a ventilator (Harvard Apparatus, South Natik, MA, USA). Subsequently, 12 rats were randomly selected as the Sham group, undergoing thoracotomy without actual LAD ligation. Following the opening of the chest and exposure of the heart, the remaining rats underwent LAD ligation using a 7.0 surgical suture near its major branching point. The existence of MI was evaluated via alterations in the electrocardiogram’s ST segment. All rat models were established successfully. Following the surgery, the rats were closely monitored. Once they were awake, the rats were subjected to subcutaneous injection with 2 mg/kg meloxicam (150,252,467, Qilu Animal Health Products Co., Ltd., Jinan, Shandong, China) for 3 consecutive days for analgesia, and intramuscular injection with 200,000 U/d penicillin sodium (H37021950, Ruiyang Pharmaceutical Co., Ltd., Zibo, Shandong, China) for 3 consecutive days for prevention of postoperative infections. After 24 h of the surgery, rats in the Sham group were all alive, while 8 rats in the MI group died and 60 survived, with a survival rate of 88.2%.

### Animal grouping and administration

MI rats were randomly assigned into the following five groups (*n* = 12): the MI group (MI rat models were established by conventional ligation of left anterior LAD [31]), the MI + IT group [MI rats were intervened by IT (5 times/week for continuous 6 weeks)], the MI + IT + SRI-011381 group [MI rats were intervened by IT 5 times per week for continuous 6 weeks and intraperitoneally injected with 30 mg/kg SRI-011381 hydrochloride dissolved in saline containing 10% dimethyl sulfoxide (DMSO) and 40% PEG300, once every two days for continuous one week [32, 33]], the MI + IT + Vector group [MI rats were intervened by IT (5 times/week for continuous 6 weeks) and intraperitoneally injected with equal amount of saline containing 10% DMSO and 40% PEG300 [33], once every two days for continuous one week], the MI + IT + Nigericin group [MI rats were intervened by IT (5 times/week for continuous 6 weeks) and gavaged with 20 mg/kg Nigericin (once a day for continuous one week) [34]]. All medications were administered 1 week after MI modeling and at the beginning of the IT program. SRI-011381 hydrochloride (HY-100,347 A) was an activator of the TGF-β1 pathway and Nigericin (HY-100,381) was an NLRP3 activator, which were all purchased from MedChemExpress (Monmouth Junction, NJ, USA); DMSO (ST038, Beyotime, Shanghai, China) and PEG300 (HY-Y0873, MedChemExpress) werethe solvents for SRI-011381.

Cardiac function and hemodynamics were examined on the second day after IT. Subsequently, the rats underwent euthanasia via overdose of intraperitoneal injection with 5% sodium pentobarbital (100 mg/kg), with their heart samples collected. The heart tissues of 6 rats in each group were subjected to hematoxylin and eosin (H&E) and Masson staining, and those of the other 6 rats were subjected to protein extraction for Western blot and ELISA assays. The experimental design process is shown in Supplementary Fig. 1.

### The determination of maximal oxygen consumption (VO_2max_)

The MI rats underwent a one-week adaptation period (the treadmill speed was increased from 5 to 10 m/min per session, and the training duration progressed from 5 to 10 min). The VO_2max_ was measured by analyzing expired gas during a progressive exercise ramp program, with 5 m/min increments every 5 min and no grade until exhaustion [35]. Gas analysis was comducted utilizing an oxygen (S-3 A/I) analyzer (Ametek, Pittsburgh, PA, USA). The VO_2max_ was calculated utilizing the determined flow via the metabolic chamber, the expired fraction of effluent oxygen, and the fraction of oxygen in room air, as previously reported [36]. The calculated VO_2max_ was 23 m/min.

### IT program

Exercise training may contribute to altering myocardial remodeling triggered by MI and ameliorate cardiac function [7–10]. As mentioned previously [37], the rats underwent one-week rest post-LAD ligation, and a one-week adaptation period, during which the training duration progressed from 5 to 10 min and the treadmill speed was increased from 5 to 10 m/min per session. After this adaptation period, the rats were subjected to aerobic exercise on the treadmill 5 times a week for 6 weeks, utilizing the overload principle through accelerating the speed. The training program lasted for 52 min/day, consisting of an 8-minute warm-up at 10 m/min (45% of VO_2max_), a 40-minute aerobic interval exercise, and ultimately a 4-minute cool-down. The aerobic IT routine consisted of a 4-minute running at 13–18 m/min (55–75% of VO_2max_) on a treadmill, and then a 4-minute active rest interval at a decreased speed of 10–15 m/min, for a total of 5 repetitive cycle. The chosen training protocol referred to an existing study [38, 39]. All training began at 8:00 p.m.

### Assessments of cardiac function and hemodynamic indicators

All rats were intraperitoneally injected with 5% pentobarbital sodium (30 mg/kg) for anesthetization, with the left ventricle ejection fraction (LVEF) and left ventricle fractional shortening (LVFS) measured by the Philips Sonos 5500 color Doppler ultrasonography (Philips, Andover, MA, USA). Following the echocardiography, the rats were inset with multiple self-made catheters comprising a large amount of heparin saline into the left ventricle via the right common carotid artery. Then, a multi-conductive physiological recorder (Chengdu Medical Instruments, Chengdu, Sichuan, China) was employed for analyzing and recording hemodynamic indicators such as left ventricular systolic pressure (LVSP), left ventricular end-systolic pressure (LVEDP), and positive and negative derivates (dP/dt max/min) [40].

#### Histological staining

The rat heart was fixed in 4% paraformaldehyde for 48 h, sequentially placed in 70% ethanol for 3 min, 95% ethanol for 3 min, anhydrous ethanol for 3 min, anhydrous ethanol for 2 min, xylene for 2 min, and xylene for 3–5 min for dehydration, and finally embedded in wax. The embedded tissues were cut into 5 µM thick continuous slices using a fully automatic vibration slicer (VT1000S, Leica, Nussloch, BW, Germany), and stored at room temperature. For the purpose of historical analysis of heart issues, H&E staining was carried out using the H&E staining kit (Solarbio, Beijing, China) following the instructions provided by the manufacturer. The pathological changes of rat heart tissues were observed and photographed under an optical microscope (Olympus Corporation, Tokyo, Japan). To assess the extent of myocardial fibrosis, Masson’s trichrome staining was conducted. According to the instructions of the Masson staining kit (AWI0267a, Abiowell, Changsha, Hunan, China), the fibrotic changes of rat heart tissues were observed under an optical microscope and documented. The average ratio of fiber area to the entire cross-sectional area of the left ventricular was determined by the stained sections, known as the percent fiber area via ImageJ (1.61 version, NIH Image, Bethesda, MD, USA) [41].

### Co-immunoprecipitation analysis

The binding between TGF-β1 and its receptor (TGF-β receiver II) was assessed by co-immunoprecipitation. On the basis of the instructions (Thermo Fisher, Waltham, MA, USA), myocardial tissues were lysed using lysis buffer [50 mM Tris-HCl, 5 mM ethylenediaminetetraacetic acid, 150 mM NaCl, 0.5% (vol/vol) Nonidet-P40 and 10% (vol/vol) glycerol, pH 7.4], and supplemented with a mixture of complete protease and phosphatase inhibitor (50X, No. P1049, Beyotime). Next, with the myocardial tissue homogenate (30 µg) serving as the input, the remaining homogenate was divided into two equal parts, which were incubated with TGF-β1 antibody (1:200, #141,302, BioLegend, Beijing, China) and corresponding immunoglobulin G (IgG) control overnight at 4 °C under gentle rotation. Protein G magnetic beads (Invitrogen, Carlsbad, CA, USA) were introduced to the lysis product, followed by incubation for 2–3 h. The precipitate was washed 5 times with tris-buffered saline in Tween-20 (TBST) buffer solution, and boiled in 1 × sodium dodecyl sulfate (SDS) sample buffer for 5 min for protein elution, which was then analyzed by Western blot.

### ROS content determination

According to the provided instructions, the ROS content in myocardial tissues was assayed using a ROS determination kit (HR8820, Biolab, Beijing, China), followed by observation and imaging using a fluorescence microscope (Leica Microsystems, Wetzlar, Germany).

### Antioxidant enzyme activity and malondialdehyde (MDA) level assessment

The activities of antioxidant enzymes [total superoxide dismutase (SOD) (S0101), catalase (CAT) (S0051) and glutathione peroxidase (GSH-Px) (S0056)] and level of lipid peroxidation product MDA (S0131) in rat myocardial tissues were measured using kits (Beyotime) in strict accordance with the instructions [42].

### Western blot

Total protein was extracted using radioimmunoprecipitation assay lysis buffer (Beyotime) containing protease inhibitors (Roche, Complete Mini, Basel, Switzerland) and quantified using a bicinchoninic acid kit (Beyotime). Then, 50 µg protein was loaded onto 10% SDS-polyacrylamide gel electrophoresis and transferred onto polyvinylidene fluoride membranes (Millipore, Billerica, MA, USA). At room temperature, the membranes were blocked with TBST (Beyotime) containing 5% skim milk and then respectively incubated with primary antibodies TGF-β1 (1:1000, ab315254, Abcam), NLRP3 (1:1000, ab263899, Abcam), ASC (1:1000, ab283684, Abcam), cleaved-caspase-1 (1:1000, GTX133447, GeneTex, Irvine, CA, USA), GAPDH (1:1000, ab9485, Abcam) and β-actin (1:1000, ab8227, Abcam) overnight at 4 °C, followed by washing and incubation with HRP-conjugated goat anti-rabbit IgG secondary antibody (1:2000, ab205718, Abcam) for 1 h. With GAPDH and β-action as the internal parameters, the protein band was assessed utilizing electrochemiluminescence (Seyotin, Guangzhou, Guangdong, China). ImageJ software (National Institutes of Health, Bethesda, MD, USA) was utilized to analyze Gray value.

### Enzyme-linked immunosorbent assay (ELISA)

In accordance with the instructions, the levels of inflammatory cytokines interleukin (IL)-1β (PI303) and IL-18 (PI555) in rat myocardial tissues were determined with the help of the ELISA kits (Beyotime), with the data collected using a microplate reader (Bio-Rad 680, Bio Rad, Hercules, CA, USA) [43].

### Statistical analysis

All data underwent statistical analysis and plotting using GraphPad Prism 8.01 (GraphPad Software, San Diego, CA, USA) software. The Shapiro-Wilk test method was adopted for normal distribution testing. Measurement data of normal distribution were presented as mean ± standard deviation. Independent sample *t*-test was adopted for comparisons between two groups, and one-way analysis of variance (ANOVA) was employed for comparisons among groups, with Tukey’s test used afterwards. *P* was acquired from a bilateral test, and *P* < 0.05 indicated a statistically significant difference.

## Results

### IT improved cardiac function in MI rats

**Fig. 1:**
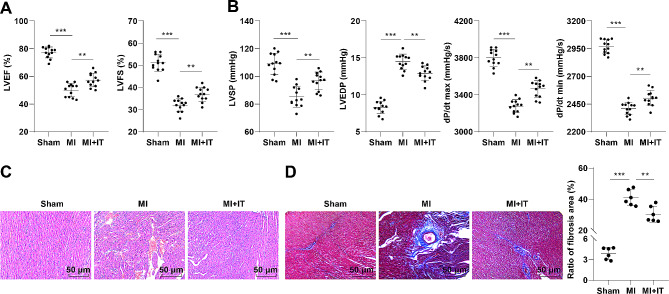
IT improved cardiac function in MI rats. **A**: Doppler echocardiography for assessing cardiac function (*n* = 12); **B**: Detection of hemodynamic indicators using a multi-channel physiological recorder (*n* = 12); **C**: H&E staining (*n* = 6); **D**: Masson staining (*n* = 6). The data were expressed as mean ± standard deviation, and analyzed by one-way ANOVA, followed by Tukey’s test. ** *P* < 0.01, *** *P* < 0.001

To preliminarily explore the role of IT in MI, we established a MI rat model and observed the effect of IT on cardiac function in MI rats. The results of Doppler echocardiography reflected that compared to the Sham group, the LVEF and LVFS of MI rats were prominently reduced, while they were raised after IT (Fig. 1A, all *P* < 0.01). The hemodynamic test results manifested that in comparison with the Sham group, the MI group displayed repressed LVSP and dP/dt max/min, and elevated LVEDP, while IT treatment brought about the opposite trends (Fig. 1B, all *P* < 0.01). As reflected by H&E staining, the myocardial tissues in the Sham group were arranged in an orderly manner and the cell morphology was normal, while the MI group exhibited a lot of scar areas in myocardium, and disordered myocardial cells; after IT intervention, the myocardial scar tissues of MI rats were distinctly reduced, and the regularity of myocardial cell arrangement and cell integrity were notably improved (Fig. 1C). The Masson staining results indicated that the myocardium of the Sham group was bright red without obvious scar areas, while the MI group appeared large areas myocardial fibrosis (blue area); after IT intervention, the area of myocardial fibrosis in MI rats was evidently decreased (Fig. 1D, *P* < 0.01).

### IT obstructed the activation of the TGF-β1 pathway to upgrade oxidative stress in myocardial tissues of MI rats

**Fig. 2:**
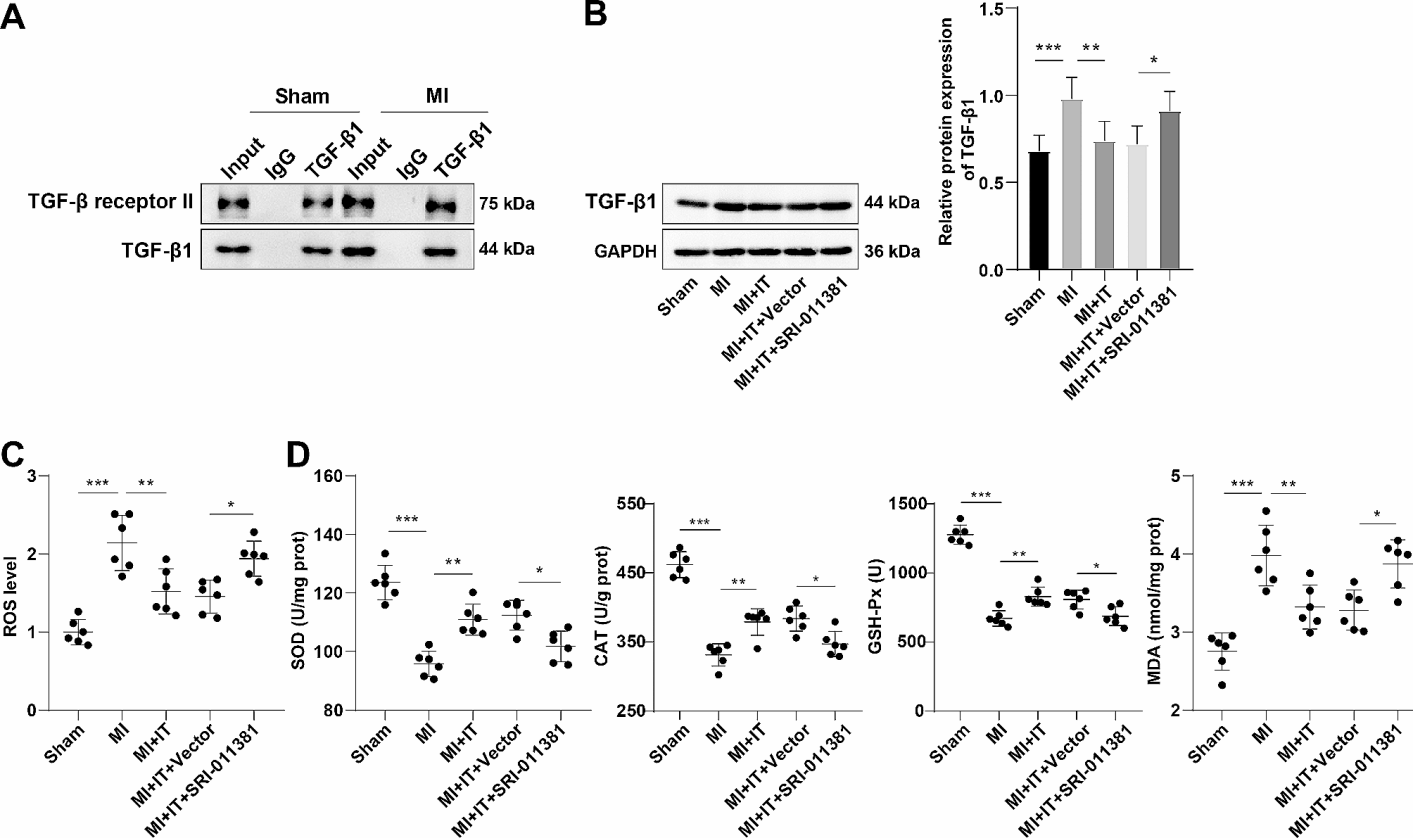
IT improved oxidative stress in myocardial tissues of MI rats by obstructing the activation of the TGF-β pathway. **A**: Co-immunoprecipitation detection of binding between TGF-β1 and receptor; **B**: The expression of TGF-β1 protein was assessed by Western blot (*n* = 3); **C**: Detecrmination of ROS content in myocardial tissues utilizing a reagent kit (*n* = 6); **D**: The kit assessed myocardial antioxidant enzyme (SOD, CAT and GSH-Px) activities and MDA level (*n* = 6). The data were expressed as mean ± standard deviation and analyzed by one-way ANOVA, followed by Tukey’s test. * *P* < 0.05, ** *P* < 0.01, *** *P* < 0.001

We conjectured that IT mitigated oxidative stress in myocardial tissues of MI rats via inactivation of the TGF-β1 pathway. Firstly, we injected a dose of 30 mg/kg the TGF-β1 pathway activator (SRI-011381 hydrochloride) intraperitoneally into rats during IT intervention, with an equivalent amount of solvent (Vector) as its negative control. The results of co-immunoprecipitation indicated that TGF-β1 and TGF-β receptor II could bind to each other, while relative to the Sham group, the MI group had increased TGF-β1 and TGF-β receptor II binding (Fig. 2A). Then, the expression of TGF-β1 protein was further determined by Western blot (Fig. 2B). The results were consistent with expectations that the MI group exhibited obviously up-regulated TGF-β1 versus the Sham group, whereas TGF-β1 was remarkablely down-regulated after IT intervention. Moreover, the MI + IT + SRI-011381 group showed prominently up-regulated TGF-β1 versus the MI + IT + Vector group (Fig. 2B, all *P* < 0.05), suggesting that IT retarded the TGF-β1 pathway activation. Furthermore, the ROS level and MDA content in the myocardial tissues of the MI group were apparently higher, while the SOD, CAT and GSH-Px activities were lower than those of the Sham group. In contrast to the MI group, the MI + IT group manifested distinctly reduced ROS level and MDA content in myocardial tissues, and a memorably increased SOD, CAT and GSH-Px activities. In addition, the MI + IT + SRI-011381 group elicited markedly higher ROS level and MDA content in the myocardial tissues, while lower SOD CAT and GSH-Px activities than the MI + IT + Vector group (Fig. 2C-D, all *P* < 0.05).

### IT extenuated cardiac function in MI rats by blocking the activation of the TGF-β1 pathway

**Fig. 3:**
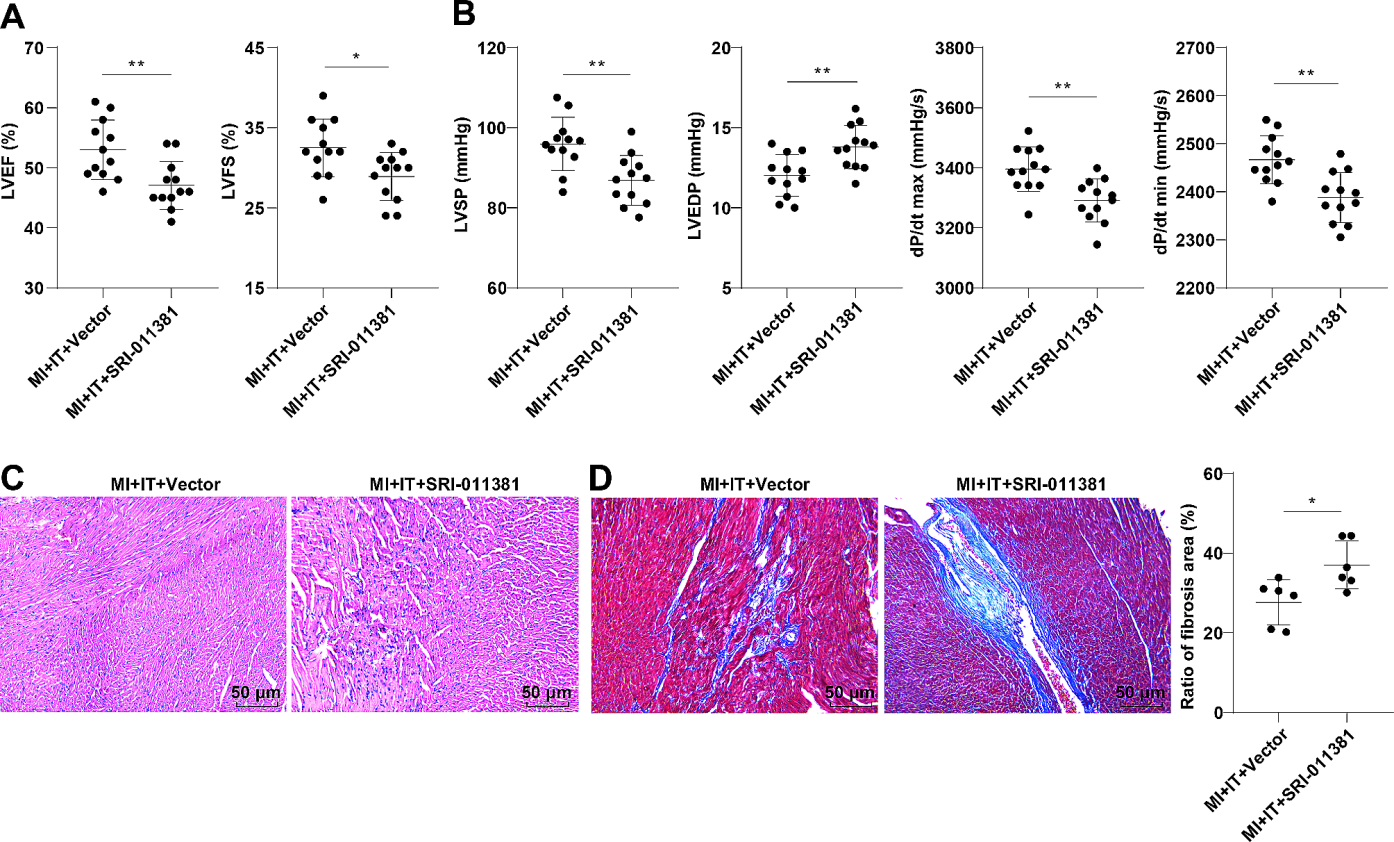
IT weakened the activation of the TGF-β1 pathway to improve cardiac function in MI rats. **A**: Doppler echocardiography for detecting cardiac function (*n* = 12); **B**: Detection of hemodynamic indicators using a multi-channel physiological recorder (*n* = 12); **C**: H&E staining (*n* = 6); **D**: Masson staining (*n* = 6). The data were expressed as mean ± standard deviation, and comparisons between two groups were analyzed by independent sample *t*-test. * *P* < 0.05, ** *P* < 0.01

To further validate whether IT ameliorated cardiac function in MI rats through hindering the activation of the TGF-β1 pathway, the alterations in hemodynamic indicators and cardiac function were assessed by doppler echocardiography and multi-conductive physiological recorder. The results demonstrated that the MI + IT + SRI-011381 group showed reductions in LVEF, LVFS, LVSP and dP/dt max/min, while an increase in LVEDP versus the MI + IT + Vector group (Fig. 3A-B, all *P* < 0.05). As shown by H&E staining and Masson staining, the MI + IT + SRI-011381 group exhibited increased myocardial scar tissues and disordered cell arrangement (Fig. 3C), and increased myocardial fibrosis areas (Fig. 3D, *P* < 0.05).

### IT allayed oxidative stress in myocardial tissues of MI rats and limited the NLRP3 inflammasome activation by impeding activation of the TGF-β1 pathway

**Fig. 4:**
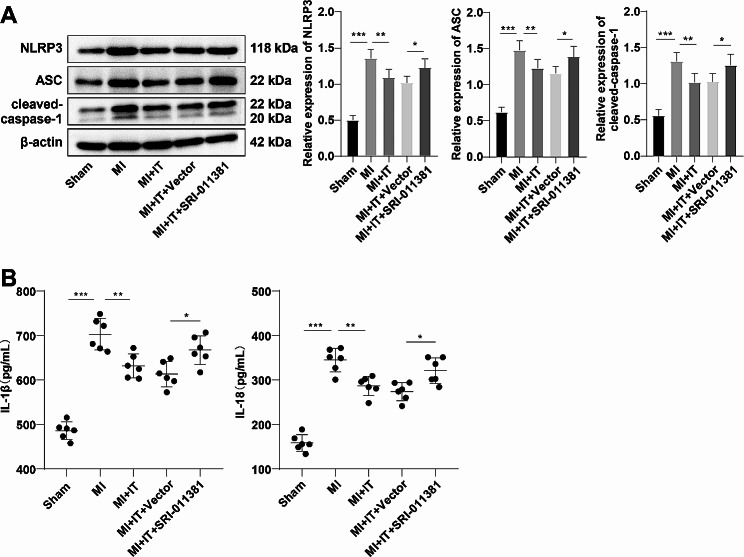
IT reduced the activation of the TGF-β1 pathway to reduce oxidative stress in myocardial tissues of MI rats and down-regulate the NLRP3 inflammasome activation. **A**: Western blot was used to determine the expression levels of inflammasome-related proteins NLRP3, ASC, and cleaved-caspase-1 (*n* = 3); **B**: ELISA to measure inflammatory factor IL-1β and IL-18 levels in myocardial tissues (*n* = 6). The data were expressed as mean ± standard deviation, with one-way ANOVA for intergroup comparisons, followed by Tukey’s test. * *P* < 0.05, ** *P* < 0.01, *** *P* < 0.001

Reportedly, oxidative stress has the ability to provoke activation of the NLPR3 inflammasomes [27], and NLRP3 inflammasomes are closely related to MI [28, 29]. To investigate whether IT improved oxidative stress in myocardial tissues of MI rats by limiting the TGF-β1 pathway activation, thereby repressing the NLRP3 inflammasome activation, we assessed the expression levels of NLPR3 inflammasome-related proteins (NLRP3, ASC, cleaved-caspase-1). As reflected by Western blot, NLRP3, ASC and cleaved-caspase-1 were markedly up-regulated in the myocardial tissues of MI group versus the Sham group, while the levels were conspicuously down-regulated in the MI + IT group; relative to the MI + IT + Vector group, the MI + IT + SRI-011381 group showed observably up-regulated NLRP3, ASC, and cleaved-caspase-1 levels in the myocardial tissues (Fig. 4A, all *P* < 0.05). Moreover, ELISA demonstrated that relative to the Sham group, the myocardial tissue IL-1β and IL-18 levels in the MI group were significantly up-regulated, whereas the levels were diminished in the MI + IT group. IL-1β and IL-18 levels were observably raised in the MI + IT + SRI-011381 group versus the MI + IT + Vector group (Fig. 4B, all *P* < 0.05).

### Activation of NLRP3 partially reversed IT-mediated ameliorative effect on cardiac function in MI rats

**Fig. 5:**
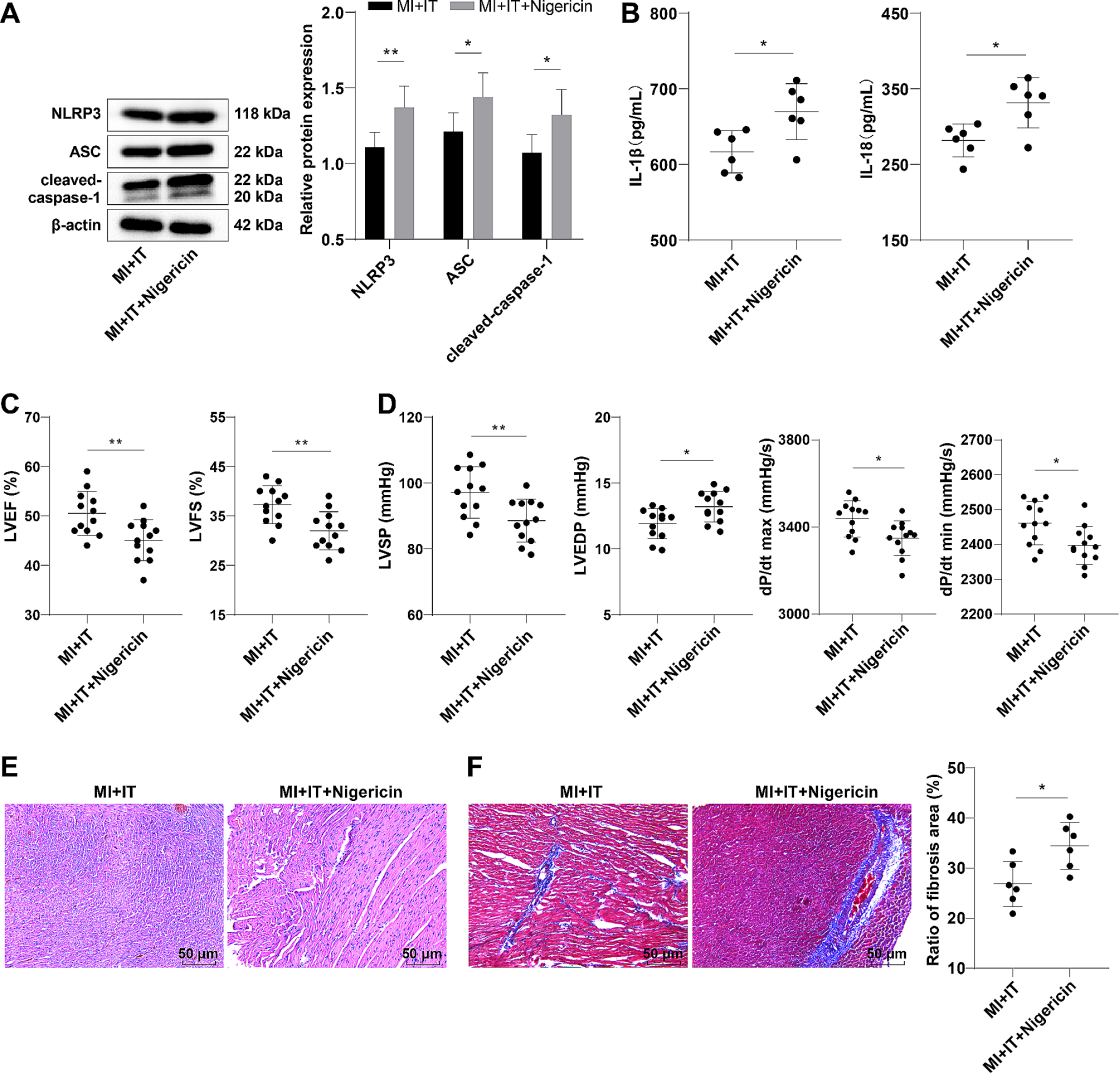
Activation of NLRP3 partially abrogated the improvement of IT on cardiac function in MI rats. **A**: Western blot was utilized to assess the expression levels of inflammasome-related proteins NLRP3, ASC, and cleaved-caspase-1 (*n* = 3); **B**: ELISA determination of inflammatory factor IL-1β and IL-18 levels (*n* = 6); **C**: Doppler echocardiography for detecting cardiac function (*n* = 12); **D**: Detection of hemodynamic indicators using a multi-channel physiological recorder (*n* = 12); **E**: H&E staining (*n* = 6); **F**: Masson staining (*n* = 6). The data were represented by mean ± standard deviation and independent sample *t*-test was used. * *P* < 0.05, ** *P* < 0.01

Next, we employed Western blot and ELISA to assess the expression patterns of the NLRP3 inflammasome-related proteins and inflammatory factors in the myocardial tissues of rats. The results declared that the levels of NLRP3, ASC and cleaved-caspase-1 proteins, and IL-1β and IL-18 of the myocardial tissues in the MI + IT + Nigericin group were higher than those in the MI + IT group (Fig. 5A-B, all *P* < 0.05). Further testing of cardiac function and hemodynamic indexes disclosed that in contrast to the MI + IT group, the LVEF, LVFS, LVSP and dP/dt max/min of the MI + IT + Nigericin group were decreased, but LVEDP was increased (Fig. 5C-D, all *P* < 0.05). The H&E staining and Masson staining results revealed that compared to the MI + IT group, the MI + IT + Nigericin group showed obviously increased myocardial scar tissues, disordered cell arrangement (Fig. 5E), and significantly increased myocardial fibrosis areas (Fig. 5F, *P* < 0.05).

## Discussion

MI is a the life-threatening coronary-related desease with high mortality globally, which is characterized by sudden cardiac death [44]. Accumulating evidence from clinic and animal research has demonstrated that exercise training is useful in ameliorating cardiac function [18, 37, 45, 46]. Besides, exercise can be beneficial to cardiac function, with an effectively preventive effect on skeletal muscle atrophy in MI patients [47]. Notably, IT has been well-documented to play a pivotal role in improving cardiac function in patients with chronic obstructive pulmonary disease [48]. In the light of this, our findings highlighted that IT diminished oxidative stress in myocardial tissues of MI rats and suppressed the activation of NLRP3 inflammasomes by hindering the TGF-β1 pathway activation, consequently extenuating the cardiac function of MI rats.

Acute MI has been found to result in marked changes in cardiac functions and structure, causing left ventricular remodeling and following diastolic and systolic dysfunction [49]. Heart failure is featured by the forming of cardiac interstitial fibrosis, and embodying the upgradation of α-smooth muscle actin, collagen I, TGF-β and collagen III [50–52]. Furthermore, cardiac hemodynamics are impaired in rats with chronic heart failure, which is manifested as elevated LVEDP, and decreased LVSP and + dp/dtmax [53]. Unsurprisingly, our findings also elicited hemodynamic damage, manifested by reductions of LVSP and dP/dt max/min and increase of LVEDP, and cardiac structure in MI rats, which were momentously extenuated via IT intervention. Similarly, high-intensity IT ameliorates cardiac function in fit rats [54]. The use of high-intensity IT contributes to obvious enhancement in prognostic cardiopulmonary exercise test parameters relative to usual care, which is associated with favorable ventricular remodeling on certain echocardiographic parameters of left ventricular function [55]. To conclude, IT may ameliorate cardiac function in MI rats.

It is also noteworthy that oxidative stress during ischemia/reperfusion or in heart failure happens as a result of the excessive accumulation or production of free radicals or their oxidation products, which plays a dominant role in post-MI left ventricular remodeling [18, 56]. Besides, TGF-β is a a critical factor in the progression and development of left ventricular remodeling and failure following MI [21]. The inhibition of TNAP attenuates MI-triggered cardiac fibrosis via the TGF-β1/Smads pathway inactivation and the P53 pathway activation [57]. Our findings manifested activated TGF-β1 pathway and facilitated oxidative stress in MI rats, which were partially reversed through IT. However, the TGF-β1 pathway activator partially averted the role of IT treatment on inactivating the TGF-β1 pathway, reducing oxidative stress, and improving cardiac functions and myocardial structure in MI rats. Moreover, aerobic IT can notably limit inflammatory reaction and myocardial oxidative stress, as well as upgrade cardiac function in MI rats, while its mechanism is closely relevant to the activation of the SIRT1-Nox4-ROS pathway [58]. High-intensity IT has also been demonstrated to relieve liver fibrosis induced by ketogenic diet in type 2 diabetic mice through the TGF-β1/Smads amelioration [59]. Furthermore, exercise training makes cardiac fibrosis in remission through controlling the TGF-β1-Smad2/3-MMP2/9 and increasing fibroblast growth factor 21 in MI mice [24]. We concluded that IT hindered the activation of the TGF-β1 pathway to improve cardiac function and oxidative stress in myocardial tissues of MI rats.

Importantly, oxidative stress has the capacity to induce activation of the NLPR3 inflammasomes [27], which is closely related to MI [28, 29]. The role of NLPR3 inflammasomes has also been identified through that miR-703 and miR-133b weaken hypoxia injury and pyroptosis by inhibiting NLRP3/caspase-1 following MI [60]. Besides, in MI experimental models, release and induction of the pro-inflammatory cytokines including IL-1 are steadily described [61–63]. We elaborated activated NLPR3 inflammasomes and elevated levels of inflammatory cytokines in MI rats, which were subsequently improved by IT, but they were aggravated again by the TGF-β1 pathway activator. What’s more, the treatment of NLRP3 activator also brought out the consistent trends with the TGF-β1 pathway activator, which abrogated the effects of IT on repressing the NLPR3 inflammasome activation, improving hemodynamics and cardiac functions along with structure. In a similar light, STING-IRF3 accelerates lipopolysaccharide-induced cardiac dysfunction, pyroptosis, apoptosis as well as inflammation via the activation of NLRP3 [64]. Activation of NLRP3 inflammasome by interaction between brain and heart, triggers cardiac inflammation and hypertrophy when pressure was overloaded [65]. Silica nanoparticles lead to cardiac hypertrophy and pyroptosis by the NLRP3/ROS/caspase-1 pathway [66]. These evidences suggested for the first time that NLRP3 activation partially reversed the improvement effect of IT on cardiac function in MI rats.

In summary, this study supported that IT obstructed the TGF-β1 pathway activation to reduce oxidative stress in myocardial tissues of MI rats, and down-regulate the activation of the NLRP3 inflammasomes, thus improving the cardiac function of MI rats, which provided some theoretical basis for clarifying the pathogenesis of MI and novel ideas for MI therapy. Nevertheless, we only established an MI rat animal model and utilized echocardiography, hemodynamics, and H&E and Masson staining to preliminarily explore the mechanism of how IT impeded the TGF-β1 pathway, improving oxidative stress in myocardial tissues of MI rats and inhibiting the NLRP3 inflammasome activation, and thus improving cardiac function in MI rats, but did not quantitatively analyze the improvement effect of IT on MI area in MI rats by TTC staining, or conduct in vitro cell experiments to further verify and explore the deeper regulatory mechanisms, which will be further refined in subsequent studies. In addition, the role of IT may vary depending on the stage and severity of MI, and the MI rats in this study may be in the acute and subacute stages. However, we did not continuously monitor the cardiac function of MI rats during the 6-week IT program, which will be further investigated in the future.

### Electronic supplementary material

Below is the link to the electronic supplementary material.

Supplementary Material 1

Supplementary Material 2

Supplementary Material 3

Supplementary Material 4

## Data Availability

All data generated or analysed during this study are included in this article. Further enquiries can be directed to the corresponding author.
