# Supplementary figures and images for: Interval training suppresses nod-like receptor protein 3 inflammasome activation to improve cardiac function in myocardial infarction rats by hindering the activation of the transforming growth factor-β1 pathway
Source: J Cardiothorac Surg. 2024 May 10;19:283. doi: 10.1186/s13019-024-02756-1 (PMC11088074; doi:10.1186/s13019-024-02756-1)

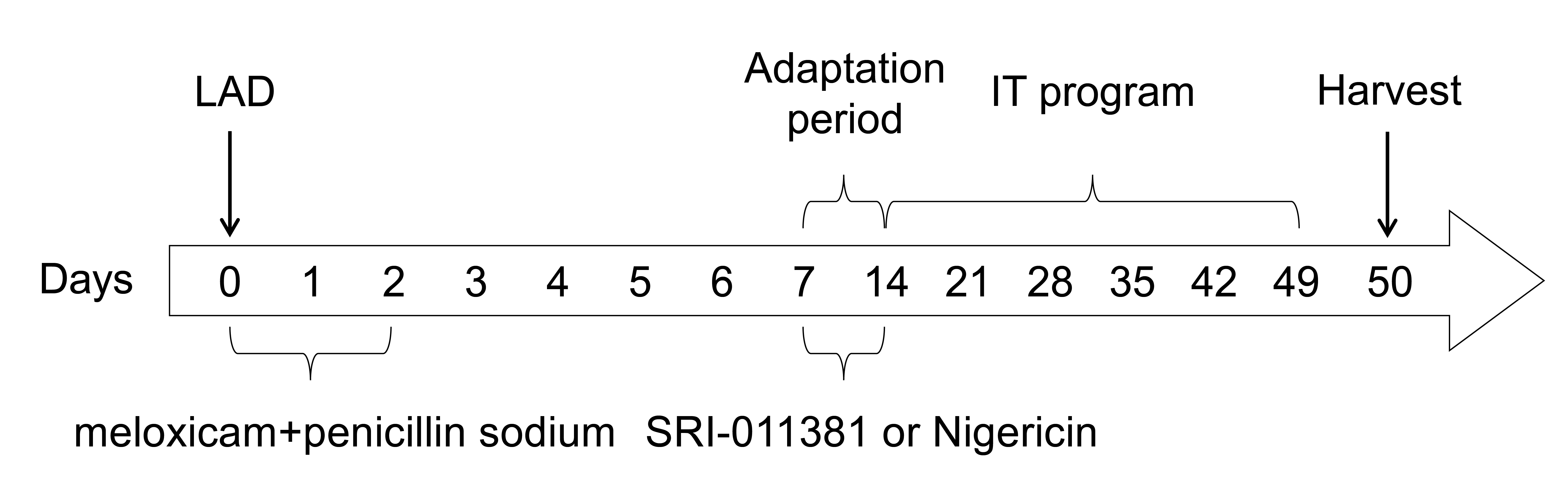

Supplement: Supplementary file 1 — Supplementary Material 1 [file 13019_2024_2756_MOESM1_ESM.png]

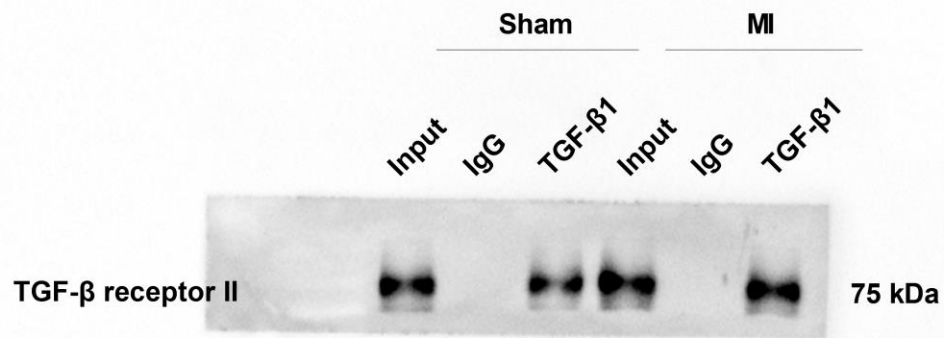

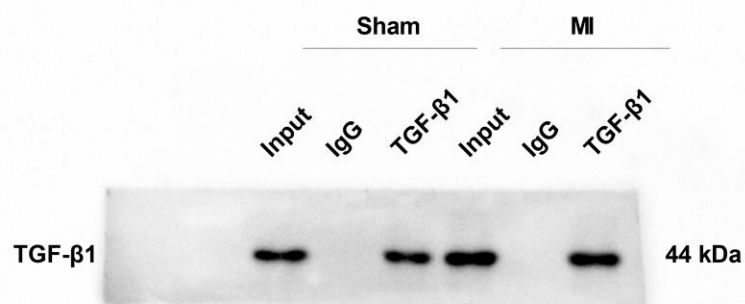

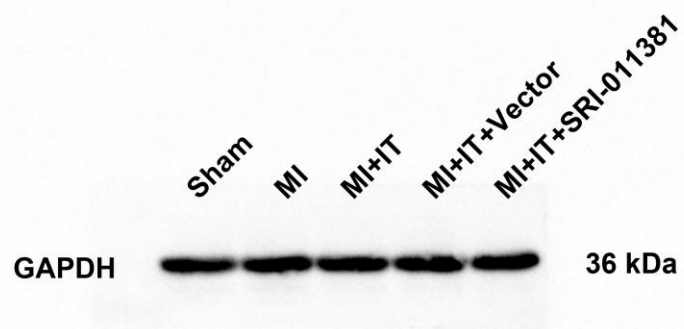

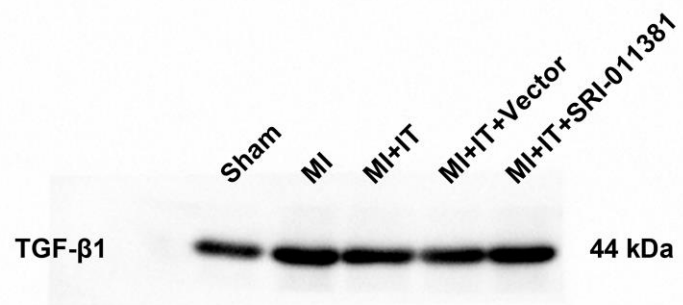

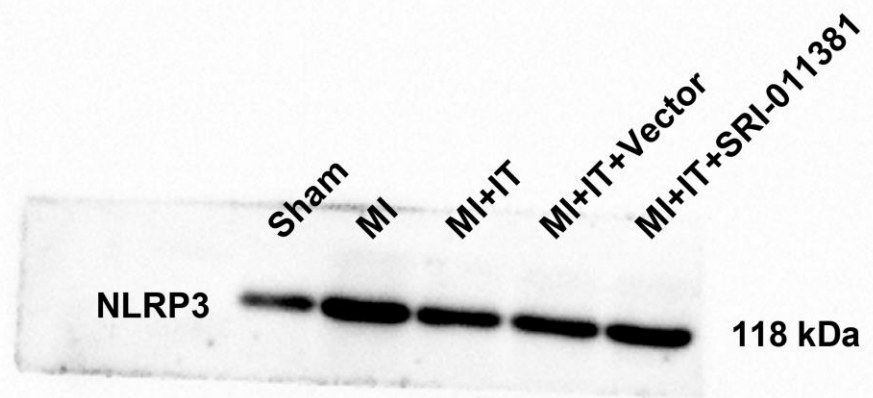

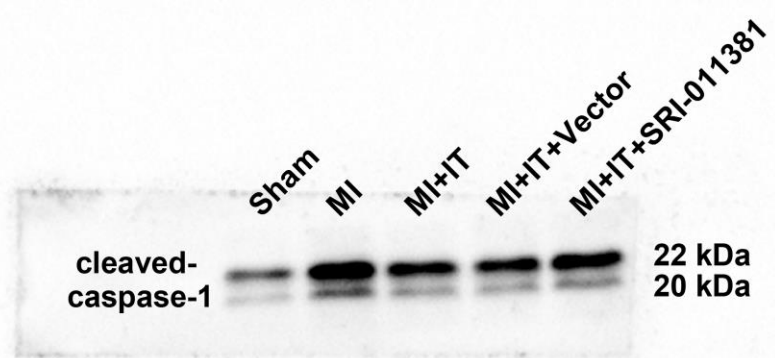

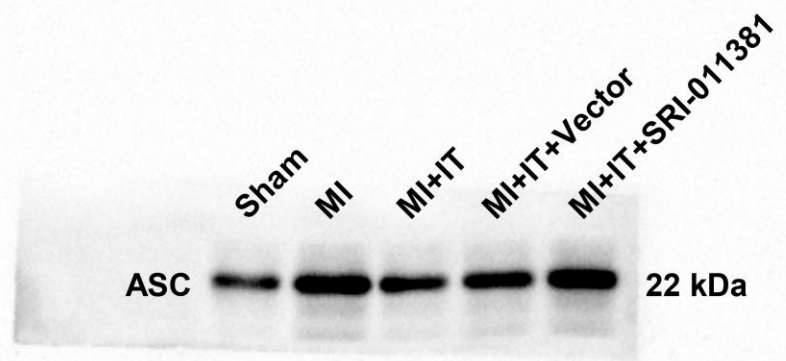

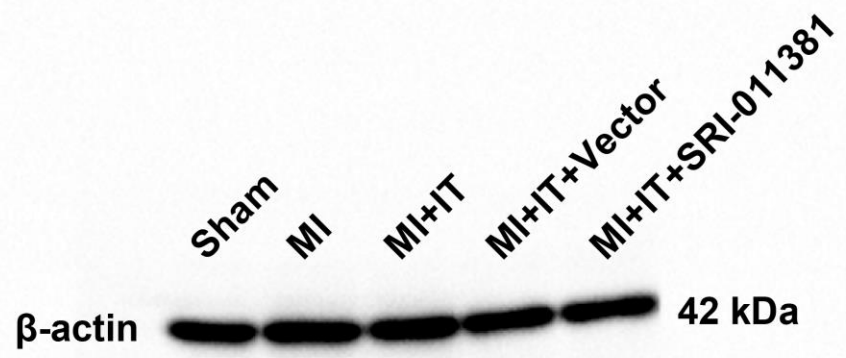

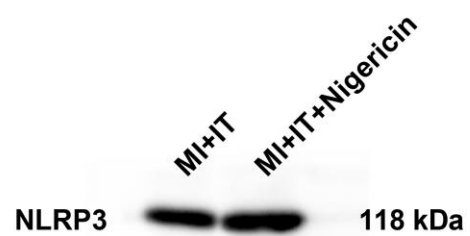

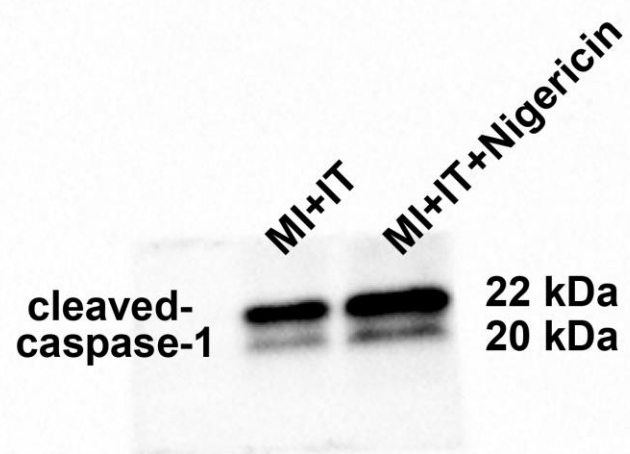

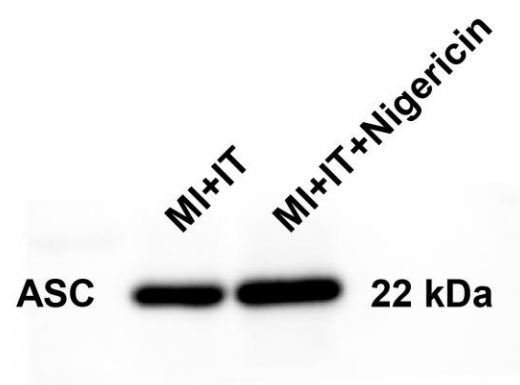

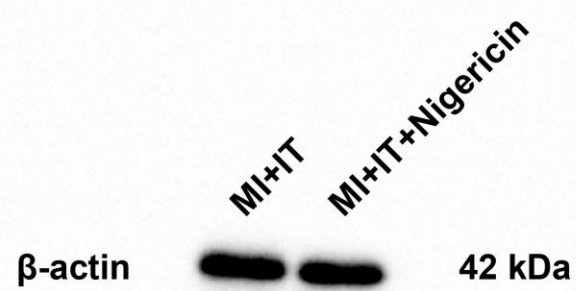

Supplement: Supplementary file 2 — Supplementary Material 2 [file 13019_2024_2756_MOESM2_ESM.pdf]

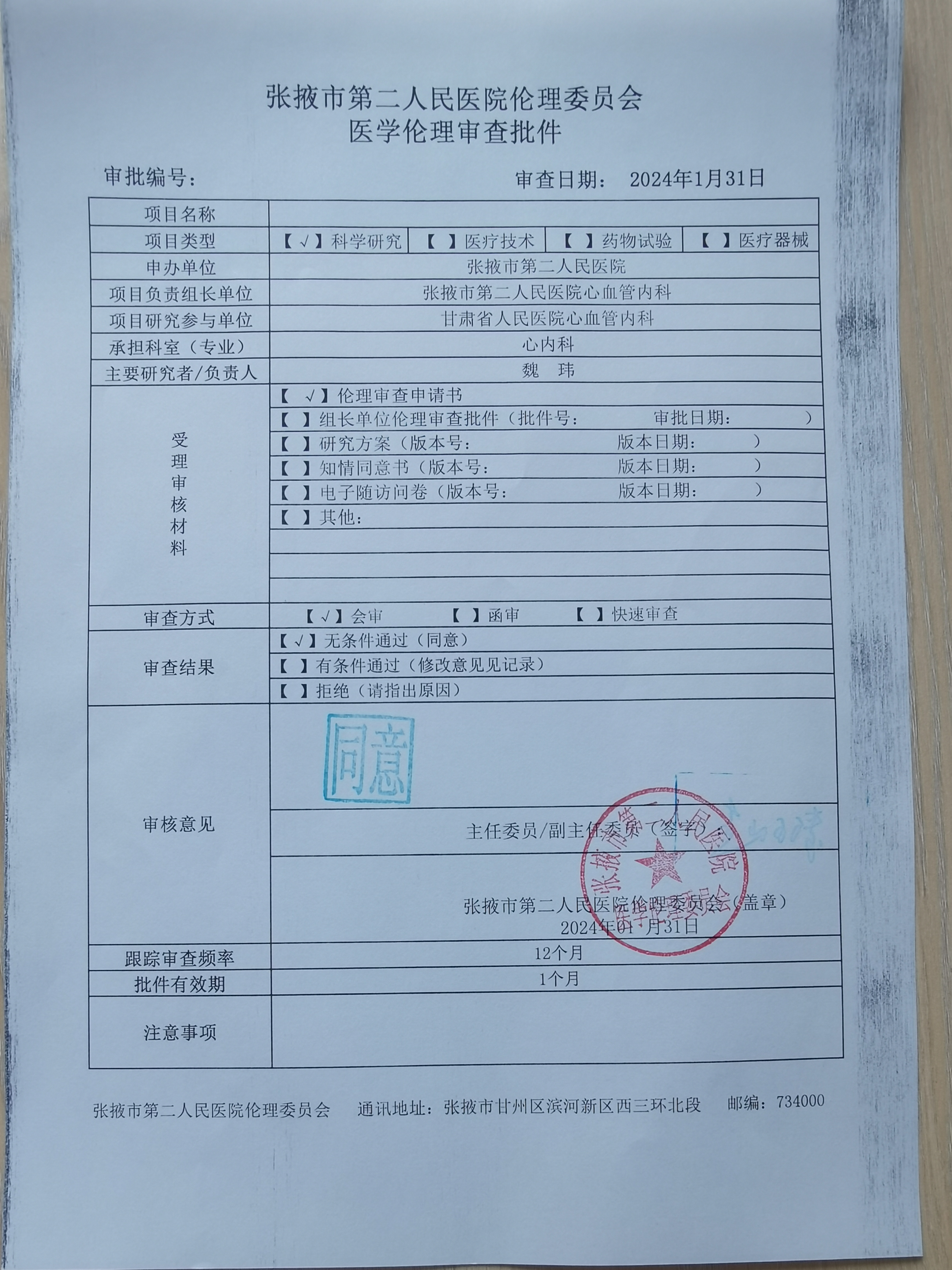

Supplement: Supplementary file 3 — Supplementary Material 3 [file 13019_2024_2756_MOESM3_ESM.jpg]

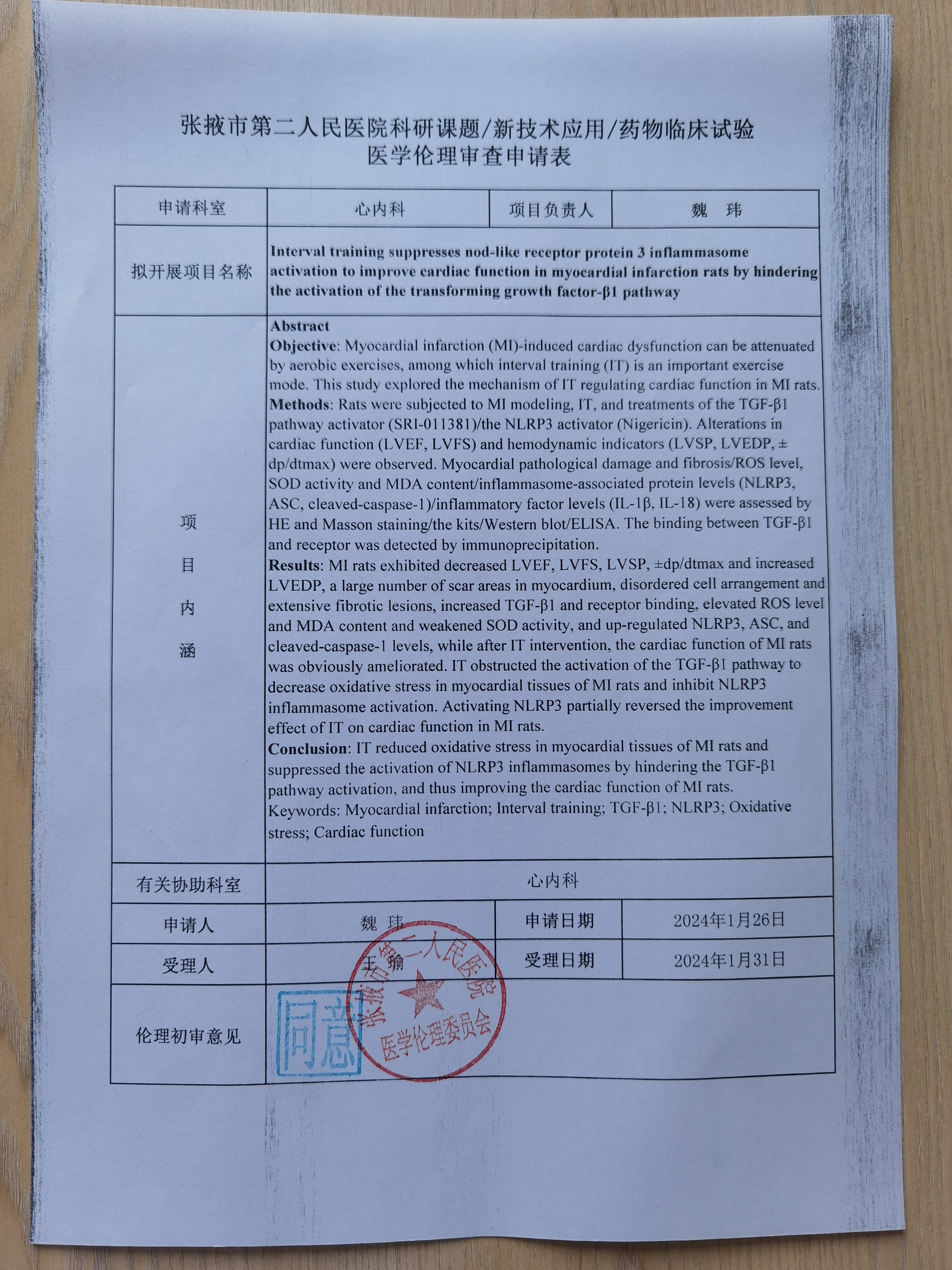

Supplement: Supplementary file 4 — Supplementary Material 4 [file 13019_2024_2756_MOESM4_ESM.jpg]
